# Factors that Influence the Effectiveness of Sanitation Programs

**DOI:** 10.3389/fpubh.2015.00201

**Published:** 2015-09-01

**Authors:** Marilu Fernandez-Haddad, Maia Ingram

**Affiliations:** ^1^Department of Marketing, Universidad de las Americas-Puebla, Cholula, Mexico; ^2^College of Public Health, University of Arizona, Tucson, AZ, USA

**Keywords:** social marketing, promotion, cleanliness of cities, new habits, public health

## Abstract

Local governments in both Mexico and the U.S. spend considerable money on public services, which do not always bring the expected results. For instance, a large part of the public budget is destined to solve social and health problems, such as public sanitation. Government has attacked the problem by providing public sanitation infrastructure (such as garbage and recycling receptacles) and by using social ad campaigns. However, these efforts do not always affect the habits of residents and bring the desired changes in city sanitation. This article presents a case study that used a participatory method to address an innovative city sanitation effort: The Clean City Program in Puebla, Mexico. This program adopted social marketing techniques, a discipline born in the 70s when the principles and practices developed to sell products and services started to be applied to sell ideas, attitudes, or behaviors. Social marketing programs have been adopted by governments to change attitudes and behavior in areas such as public services. The article first describes the context and strategies of the program, which included the use of the *promotora model* to engage community members. The researchers then make use of qualitative data gathered throughout program planning and implementation to evaluate the impact of the social marketing programs and its effectiveness. The article analyzes social, educational, economic, demographic, and cultural factors that influence the effectiveness of sanitation programs and presents recommendations for strategies to engage community members in community sanitation programs.

## Introduction

Local governments and nonprofit civil organizations in both Mexico and the U.S. have found it necessary to implement social programs that benefit different sectors of the population in order to generate social welfare and public values. These social programs are intended to solve problems in different areas such as health, security, or environment, conducting work and providing economic resources for the operation of these programs. Despite great effort, on occasion, they do not bring the expected change.

In the municipality of Puebla, Mexico, the organization responsible for operating sanitation cleaning services (OOSL) collected between 1650 and 1700 tons of garbage from private houses daily. In 2008, OOSL (1) used 60% of its budget for this garbage collection process. In addition, sanitation workers manually pick up 105 tons of garbage every day, utilizing 25% of the total budget. Given the amount of garbage in city streets, OOSL hires around 360 manual garbage collectors, who cover 2.5 km per shift under the best of weather conditions in order to solve the city litter problem and achieve a cleaner city. Therefore, the government of Puebla, during the period 2008–2010, created the program “Puebla Limpia,” the first social marketing program directed at this social problem.

The program “Puebla Limpia” promoted sanitation habits in the city: put trash where it belongs, clean and paint the facades of your home, and take trash out only the day and hour that the garbage truck comes to collect it. However, these new habits had personal monetary and nonmonetary costs that increased or decreased the likelihood that citizens would adopt them. The program used mass media to increase the level of knowledge of the program, and a direct media campaign initiated by *promotoras de limpieza* provided a mechanism of persuasion to change the habits of the residents. We analyzed and evaluated the behavior of the inhabitants of the city of Puebla in order to understand if these sanitation programs were using the right concepts to convince residents to keep the city clean.

### Theoretical grounding of social marketing

Nowadays, every society has to face and solve diverse social problems, such as alcoholism, drug addiction, degenerative chronic illnesses, and environmental pollution, which can be managed in different ways. One approach to address these issues is social marketing, which is designed to promote modifications of behavior that benefit the society (2), with the ultimate purpose of improving the social conditions that support quality of life. The authors Kotler et al. (3), Sargeant (4), Smith (5), and Weinreich (6) agree that in order to achieve the objectives of behavior change, the marketing mix should be applied correctly, which is defined by the four P’s: developing the right product, supported by the right promotion, and put in the right place at the right price.

With respect to the “social product,” the product must be described in the program as the selection of an idea, attitude, behavior, or service (product) that intends to be adopted by individuals. Social products can be challenging because they cannot be packed and do not have a monetary price; however, they are still considered a product, although not tangible. It should be affirmed that the social product has benefits (5).

Price “is the consideration of the cost–benefit to the audience.” “The price of a social product is the cost that the beneficiary associates with the adoption of the new behavior” [(3), p. 217]. In other words, what the consumer will sacrifice or pay if they adopt a new behavior. This cost can be monetary or nonmonetary. Monetary value refers to the price of the products or services to support the behavior change. Nonmonetary costs are intangible aspects such as time, effort, and energy that are implied to develop a behavior as well as perceived psychological risks, physical discomforts, and/or implied experience.

“Place is where and when the marketing goal will develop the desired behavior, or acquire tangible products and associated services” [(3), p. 243]. The objective of developing marketing strategies related to place is to make the acquisition of products and services that help the adoption of a behavior accessible and pleasant.

“Promotion is the coordination of all the activities that the promoter initiates to establish channels of information and convincing methods directed for the sale of goods and services or to prompt an idea” [(7), p. 16]. Promotion involves developing a strategy of communication, rooted in the creation of a message and the selection of the media through which it will be known.

According to the American Association of Advertising Agencies, Integrated Marketing Communications is defined as “A concept of planning marketing communication that recognizes the aggregate value of a complete plan in which the strategic functions are evaluated by a diversity of communication tools and which are combined to achieve the clarity, coherence, and maximum effect of the communication” [(7), p. 35]. Kotler et al. (3) propose an eight-step model to develop social marketing plan. This is the model used in the development of the *Puebla Limpia* case study.

1. The environment of social marketing.
2. Selection of target.
3. Establish goals.
4. Analysis of the audiences and competition.
5. Strategies of social marketing.
6. Develop a plan for evaluation and monitoring.
7. Establishing budget and sources of financing.
8. Implementation by step.

### Behavior change theory

Considering that the fundamental objective of social marketing is to achieve a desired behavior change, it is important to review a model of behavioral change to identify the stages in which social marketing can achieve desired modifications in behavior. The Stages of Change model was developed by Prochaska and DiClemente (8) and describes the motivation of a person and his/her disposition to change behavior. The model’s basic premise is that behavior change is a process, not an event. Since the 90s, Stages of Change has become one of the most applied models in developing social marketing campaigns to detail the stages of physical activity among the residents of a determined community (9). The model arose from the analysis of the main theories of psychotherapy and behavior modification, in which 10 different processes of change were identified. These processes suggest interventions of change that will be more appropriate and effective if they are carried out in six phases:

- Pre-contemplation: when the individual has no intention of taking action (possibly through ignorance of the problem) within the next 6 months.
- Contemplation: when the individual is seriously thinking of changing in some reasonably short time frame (6 months).
- Preparation: when the individual has formed an intention and is planning to actually assume the behavior and has taken some behavioral steps in this direction.
- Action: when the individual is clearly changing.
- Maintenance: the individual has changed behavior for more than 6 months.
- Termination: when the individual is not tempted to reengage in the old undesirable behavior (8).

## Research Design

The purpose of the study is to determine the key factors that influence the effectiveness of social marketing programs in the municipality of Puebla and to identify the determinant factors (educational, cultural, economic, social, and/or demographic) that influenced the effectiveness of the program or the adoption of habits with regard to the cleaning of the communities. The specific objectives of the study were to (1) identify residents’ perception toward the cleanliness of the city of Puebla; (2) analyze why different areas of the city of Puebla stay clean or dirty; (3) analyze a social marketing program to promote the cleanliness of the city; (4) analyze the acceptance of the promoted habits of the Puebla Limpia program; (5) compare the impact of the program in three different urban areas of the city; (6) identify the involved factors in the effectiveness of the program on those areas; and finally (7) give proposals for improvement of the next developmental stages.

Action Research (AR) provided the research methodology as proposed by Coghlan and Brannick (10). In this approach, a collaborative relationship between researcher and organization aims at both solving a problem and generating new knowledge. AR projects are often presented in the terms used to describe a case study which include (1) describing the context and purpose of the project; (2) analyzing the problem under investigation and the planning of the intervention; (3) describing how the intervention was executed; and finally, (4) evaluating the actions taken.

### *Puebla Limpia* case study

Consistent with the AR approach, the Puebla City government requested that the research partner assist them in an effort to develop the sanitation program. The researchers joined the program in a temporary facilitative role and worked with the members for the duration of the project to manage and create a method of action that promoted the desired changes. Therefore, the researchers were directly involved in the creation and planning of the program and as a part of the operating committee were responsible for developing the program. At the same time, the researchers developed the system for and recorded the measurement of what happened during the intervention in order to change or improve the functionality of some aspects of the process as well as to learn from it.

Garbage in the streets and public spaces is a problem that plagues the city of Puebla, affecting the city’s image and causing complications for its national and international promotion of tourism. As a major metropolitan area, the city carries the responsibility to improve its sanitation service infrastructure as well as to provide a greater number of trash bins in the streets. However, although the collection service exists in 97% of the districts and public areas are cleaned and swept daily, they get dirty again by citizens passing through these areas. Every day, 100 tons of garbage is manually collected from the streets.

In 2008, before starting the Puebla Limpia program, the city had 5000 waste bins citywide in strategic areas: where there is greater concentration of pedestrians, bus stops, and shopping malls, with street lamps, high banner poles or traffic lights, etc. However, the municipal government invested in 2500 more to provide a greater coverage of service. Therefore, a social marketing program was designed to trigger changes in the inhabitants of Puebla and break the social norms with respect to cleaning habits and thus reduce the trash in public places. We now describe how the components of social marketing were applied in the program.

#### Product

In order to create a tangible social product (3), the city developed a Social Co-Responsibility Pact, a short document with cleaning habits to implement, which the citizens promised to undertake through their signature. To reinforce the pact, they were given a badge with the words “I love a Puebla Limpia” that would identify them as a program member and a partner citizen committed to cleaning their environment.

#### Price

Considering the adoption of new habits, such as cleaning common areas, is not an easy subject. It was decided to create monetary incentives for citizens who want to participate in the public events to clean common areas.

#### Place

As with tangible products, ideas, habits, and behaviors should be available in different places for the population, and so the distribution of the program took place throughout the city through three distribution channels: first, define the points to clean through Cleaning Marathons; second, identify the distribution modules for the Social Pact for Co-Responsibility “I love a Puebla Limpia”; and the third channel was the promotoras who organized multilevel awareness-raising meetings in homes.

#### Promotion

The promotion of Puebla Limpia was developed through a campaign in both mass media and “below the line” media or messaging that takes place through less conventional avenues. In the case of *Puebla Limpia*, *promotoras* conveyed the messages within the community setting. The messages in the first phase were focused on conscientiousness and were positive, happy, and appealed to people’s participation with the implementation of concrete actions: habits of cleanliness. However, in the second phase of the program, the message was tougher, with fines or penalties, to make people reflect on what might happen if citizens continue having bad habits, and moving them to react to this stronger message.

#### Partnership

The city developed a Social Investment Portfolio, which functioned as a link between city government, businesses, and NGOs that wanted to join the program. It also invited the mass media to participate as spokespersons of the program as well as the leaders of different districts and community presidents, school principals, business leaders, among others.

#### Policy

An operating committee was established for Puebla Limpia, which presented the program with the ultimate goal of state agencies adopting it as their own and actively participating in the operation and promotion of the program. The committee integrated representation from all sectors of city government coordinating specific activities for its operation.

### Study design

Two stages of intervention were carried out by the Puebla Limpia program at two different times. Therefore, the first observation was made with no intervention (O1) in May 2008; then the first intervention (X1) with messages about the importance of conscientiousness adopting four habits for a clean city, which lasted from June to September 2008; later in September 2008, the second observation (O2) was performed to identify changes in perceptions and behavior of individuals after the first intervention. One year later from July to November 2009, the second intervention (X2) was performed, changing the marketing mix, with an emphasis on fines and penalties. Adjustments were made according to the assessment, which took into account the new information and thereby initiated a new cycle of diagnosis, planning, and AR. Finally, the third observation (O3) was made to identify final behavioral changes.


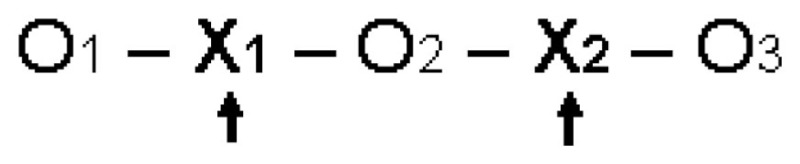


In this sense and considering that social marketing is an underutilized tool, the investigation was initiated as an exploratory investigation for the purpose of gaining an understanding of the context and elaboration of the program and thus making a diagnosis and broader case plan for urban sanitation programs. Secondary data sources and qualitative research, such as observation and in-depth interviews and focus group, were the basis of this exploratory phase.

#### Qualitative Methods

The study used theoretical sampling methods in which the boundary of the size and conformation of the sample were defined by the principle of theoretical saturation. In this approach, as the study advances, the researcher will select additional cases that consciously contribute to get useful information for the understanding of the research problem (11). In a qualitative study, it is not necessary to define the sample from the initial moment of the proposed investigation. The sample selection is flexible and is defined as the investigation progresses according to Hernández et al. (12). Qualitative methods included in-depth interviews, short interviews, focus groups, and observations.

In-depth interviews were administered to managers and operative personnel of the Cleaning Operation for Puebla government to know the previous and current situation of the problem and its plans in reference to infrastructure and services to improve the cleanliness of the city. These interviews were conducted for both the first and second interventions. The coordinators responsible for performing cleaning marathons in different parts of the city were interviewed in order to know their perceptions of the willingness of people to participate in cleaning the city. These interviews were conducted at the end of the second intervention. The interview consisted of 24 questions that addressed perspectives about the status of cleanliness in the city, its issues, operation and service results, human resources and materials, among other topics.

*Promotoras* conducted short interviews with inhabitants contacted through multilevel awareness-raising meetings. The purpose of these interviews was to identify knowledge and interest in the program before and after the meetings that were held. These interviews were conducted before the end of the first intervention. In addition to demographics, the first questionnaire determined if or where the respondent had heard of Puebla Limpia, by what means, what was the program about, and which habits they had committed to. After the meeting, the same people were asked again what it was, and how Puebla Limpia habits were undertaken, among other things, to identify changes in the perception of Puebla Limpia and change in their willingness toward the proposed habits.

Focus groups were conducted to evaluate the content of the campaign before its release. Three groups were held in 2008 and another three groups to evaluate the campaign before the launch in 2009. Participants included housewives, young people, and male workers. The focus group guide sought opinions of the cleanliness of the city, proposals for improvement, willingness to support the program, and an assessment of the 2008 campaign artistically. The discussion sought to identify reactions to the creativity, message clarity, and recall, among other things. The second focus group guide also had general questions about recall of the campaign and promoted habits, what habits they were willing to adopt, by what means they had learned the program, proposals for a second phase, and the evaluation of the Campaign 2009 artistically, to identify the target audience reaction to the creativity, message clarity, and recall, among other things.

Based on the information collected in in-depth interviews, we selected the places with larger cleaning problems for observation as well as by demographic and psychographic characteristics. Three types of places were selected: multiple family housing, low-income housing, and middle-class communities. We first used an open notebook method to register the patterns of conduct of the observed. Subsequently, a descriptive system of observation was utilized taking notes on the environment through a registry page (pages of codification), which allowed the researcher to register the most interesting data from each session of observation. The register allowed the notation of observations of dirty places, the kind of dirtiness, motives or reasons for the dirt, the kind of place, habits of the inhabitants or pedestrians, among other things.

#### Qualitative Data Analysis

The analysis of data from in-depth interviews, short interviews, and focus groups was carried out by means of literal transcription of the recordings obtained during the sessions and subsequently analyzed by identifying the most prominent items or variables and reporting them in summarizing blocks with complete sentences supported by quotations from people interviewed. As for the observations realized, the collected information in pages of codification was synthesized into a general summary, written in prose (13).

The data presented in the case study are based on program evaluation activities and do not include any human subject’s research.

## Results

To assess the effectiveness of the program, the qualitative results of the research are presented, based on observations, in-depth interviews carried out with operating coordinators of the program, *promotoras*, and focus group with inhabitants. The results are organized in eight sections, referring to the elements of social marketing as described in the literature, shown in the following model:


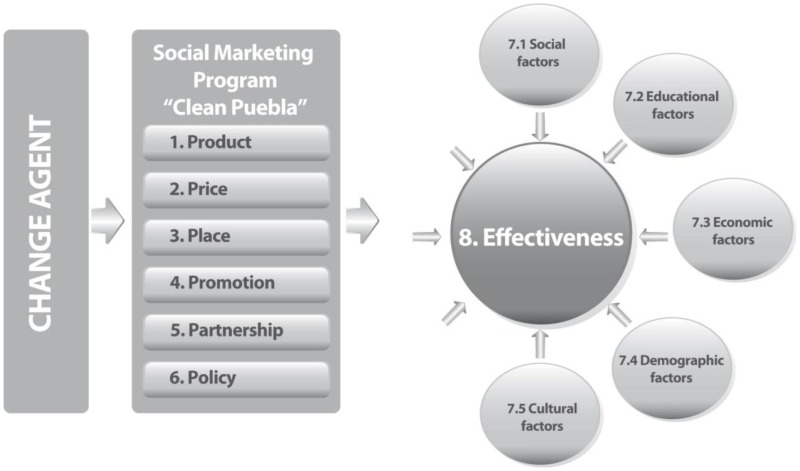


### Product (*Puebla Limpia*)

As for the perception of the program, it received acceptance among the inhabitants of the city of Puebla because they consider that Puebla Limpia was a relevant program for the city. For example, one of the program managers said that the program *“is a concept that the people accept easily as being necessary, it is a common aspiration *…* They recognize that it is not the government’s job, but for all the inhabitants of the city” (General Coordinator of Marathons for Puebla Limpia)*.

Citizens also saw value in the program. For example, one of them commented: *“It is good that this issue is being addressed *…* The true problem of the trash is that it blocks the drains and we are going to be inundated, this is truly a problem” (Maribel, 20 years old, high school education)*. After contact with the program, another resident commented: “*It interests me to know more about how we can help to have a clean city, to have my street clean, and my community clean. Because if we join the program in this, we are going to have a better city” (Patricia, 42 years old, elementary school education)*.

Older residents had memories of Puebla as a cleaner city and recognized the role of citizens in this effort.

Cleanliness interests me a lot, and I would like us to be the city that we were years ago, one of the cleanest cities, when our parents went out to sweep the street and those who did not do it were fined … for this reason there was a song “How Pretty is Puebla” (Enrique, 56 years old, technician). “To maintain a Puebla Limpia is also our responsibility, because it is the place where we live. We must be obligated to recover the values that we had before, to go out and sweep in front of our house because for many years that was not an option, it was an obligation of all the citizens …” (Mr. Flores, 50, retired.)

### Price (cost)

The four habits considered in the program were to (1) clean public spaces around homes; (2) put trash where it belongs; (3) take out the trash in closed bags only on the day the garbage truck comes to collect it; and (4) clean the facades of houses. *Puebla Limpia* results show that habits and behaviors are difficult to change because the citizens find the associated costs high since they have to invest time and money and make a physical effort in order to change their actions.

The habit of *not throwing trash* was the most accepted because it was the one that needed the least effort and had the greatest benefit *“It would be good if we all put the trash in its place, and then the drains would no longer be covered so we do not flood our roads” (Catarina, 30 years old, elementary school education)*.

The habit of *sweeping* was an action that was also considered;*“to sweep in front of my house, why not? – my cleaning reflects on me” (Ana, 23 years old, elementary school education)*. Nevertheless, sweeping in front of the house implied that people must get up earlier or that in the afternoon, they find 15 min to clean their streets: *“I need to dedicate at least 15 minutes daily, it is a lot when one is always running, besides if all the neighbors did the same thing it would be useful, because if not, the garbage is blown from one side to another”*. But they expressed frustration *“it doesn’t make sense for me to clean if the trash of the others is going to fly to my area”. (David, 45, elementary school education)*.

A nonaccepted habit by the majority of the participants from the focus group was *taking out the trash at the scheduled collection times* since they argued that there was a lack of punctuality in the routes of the garbage trucks as well as the personal impossibility to do it at the time that was on the schedule because it was to be done in the early morning or because they worked during the day. *“The truck at times does not pass at the stated time or passes in the early morning and therefore we put out the trash beforehand”*. Nevertheless, they agreed to put it in closed bags. Thus, participants had the willingness to change little by little their behaviors, but they expected that the corresponding institution or municipal departments did theirs.

*To fix the facade* was one of the habits that the people were less willing to adopt since the monetary cost was one of the factors that impeded in greater measure the change of this behavior. Besides that, this habit implied a greater amount of time for execution *“to fix my store front yes, if only to clean my windows, but to paint is a difficult situation” (María, 28, elementary school education)*. In addition, graffiti disinclined the community to paint their facade since they perceived beforehand that that effort would not be worthwhile. *“I can agree with almost all the habits. The only one that I outright cannot agree with, is that of painting, because what good is it for me to paint, if they already came and left me their autograph on the wall” (Diana, 35, middle school education.)*.

In conclusion, it took an effort for people to accept the civic habit to put out the trash just before the garbage truck passed, to paint the façade of the building, or to sweep in front of the house. This was also because of a lack of motivation, time, or money, but on the other hand, there were some people who accepted putting the trash out and taking it out in tightly closed bags because that implied a smaller effort. In view of this situation, Puebla Limpia had a program of prizes and incentives to motivate the citizens through cleaning marathons by area and motivate those habits that took effort to carry out.

### Place

The distribution of the *Puebla Limpia* activities was developed to reach all parts of the City through direct and mass media communication; nevertheless, to be able to take care of the areas that presented the greatest garbage problems, cleaning marathons were organized in nine types of areas in the City. Through observation of the nine types of areas, it was found that those areas that remained dirty or clean depended on the citizens’ demographics and were the residential category of the observed neighborhoods. Observations of public plazas, markets, schools, and large avenues lead me to the conclusion that their cleanliness depended more on the government, the director, or the administrator of the housing complex, and not from people’s habits. Therefore, we decided to focus only on three different housing zones of the city where changes in habits could be observed: housing zones of middle class (average income communities), housing zones of lower class (government housing and lower income communities), and apartment buildings or housing complexes.

The Cleaning Marathons reached 25% of the population of the city including housing complexes, government housing, and communities, in addition to the markets and schools.

Not all the inhabitants go out to clean, nor do all the places remain clean. Nevertheless the interesting thing is that many areas where constructive leadership exists, the exercise of cleaning spontaneously continues, and those places are forming the habit of having their space clean (General Coordinator for Cleaning Marathons)

This was further emphasized by a neighbor of the Prados Agua Azul Community *“Some neighbors are a little unwilling to cooperate and at times one must pressure them* …* therefore we organized the neighbors and we went house by house to motivate them to help us to clean, but I believe that it was more from the social pressure, because we were already organized. The others felt out of place if they didn’t cooperate, so they had to join *…*” (Mr. Flores, 50, retired)*.

In a similar way, Puebla Limpia reached 15,000 homes with an awareness-raising workshop house by house (direct communication). Meetings were held in different parts of the city, which had the objective of motivating small groups of the population (10–20 people) to undertake the cleaning in their community. The marathon organizers identified that after awareness workshops, attendees were motivated to participate much quicker in organizing a cleaning marathon in their area.

### Promotion

#### Mass Media Campaign

The promotion of Puebla Limpia was developed through a mass media campaign and alternative (direct) communication. The mass media campaign was developed for television, radio, print, and outdoor billboards and helped to inform the people about the importance of having a clean city. It also familiarized the people with the program and raised public awareness on the adoption of civic duty. In addition, it worked to remind people of the importance of having a clean city and to support the cleaning of different areas. *“The ads on TV or radio served to reflect on the topic, to renew a commitment to resume habits and values lost over time” (Marcela, 68, salesperson)*.

In general, the target audience heard about the campaign Puebla Limpia mainly by radio and television, and the elements of the campaign that were most often recalled more than a year into the program *I love A Puebla Limpia* were: the personalities of the campaign, the heart logo that said “I love a Puebla Limpia,” and the catchy rhythmic music as discussed in the focus groups: *“I remember the little tune with the whistle, very nice girls, but especially the heart *…* I think that is synonymous with the Puebla Limpia heart, wanting it for Puebla” (Gabriela, 27, employee). “I know the campaign on radio and TV *…* I like the girl. She is very nice and very enthusiastic, with energy”*…* I also really like the heart of “I love a Puebla Limpia”. I feel like it is to love Puebla” (Consuelo, 53, housewife)*.

In general, residents remembered that the campaign talked about avoiding making the city filthy. They acknowledged that the TV and radio gave them information on what was needed to improve the habits in order to have a clean city, and it awakened an interest in them to achieve this goal. However, the mass media did not help them to clearly and securely identify the benefits from adopting these habits of cleanliness, much less feel a commitment to make them*. “Yeah, I saw on TV that we must begin to sweep the streets and not throw garbage just anywhere” (Tomasa, 37, elementary school education). “It tries to unite us for cleaner streets, throwing trash in its proper place” (Enrique, 56, technician). “… well, what I agree with is that we ask people to no longer pollute our city” (Patricia, 46, bachelor degree)*.

Regarding the four cleaning habits that were promoted, there were two that were remembered most often and easier to adopt: to sweep in front of the house and put the trash in cans. Surely, the failure to put trash in cans was the lack of proper education of the citizens, and sweeping in front of the house is a habit that had been lost over the years.

The messages in the first phase (2008) of Puebla Limpia were oriented to raising awareness and were positive, happy, and invited people to participate and get involved in the four cleaning habits with their city. However, participants in focus groups conducted in 2008 insisted that the message should have been stricter applying fines or penalties, so that people would react to what could happen if they continued with improper habits. Therefore, the 2009 campaign addressed the issue of “zero waste, zero fines, I love a Puebla Limpia”, which served to advertise what was prohibited *“in practice this has served to admonish those who throw garbage in the wrong places, or leave trash bags in a prohibited area and to not be surprised when they are fined” (Director of Operations, Garbage Collection Service)*. The announcement of the concept was welcomed because it was reassuring to know that those who did not have good behavior at last were going to be fined or sanctioned*. “People tell us, finally fine those who throw garbage in the streets … other people basically disagree that we are so dirty” (General Coordinator for Cleaning Marathons). “It is okay to fine them, because more than anything it is a problem of culture and neglect by the people, so let’s see if this action retrains people a little to see if in this way we can do more to help clean the city” (Blanca, 22, salesperson)*. Sometimes human beings have to feel threatened to take action when desiring behavioral change, we grow up socially conditioned to the good and bad consequences our actions may have.

#### Alternative and Direct Media Campaigns

The campaign had also been developed through direct means with *promotoras* conducting over 15,000 house-to-house meetings going from house to house to persuade nearly 300,000 inhabitants in the three residential areas to change their habits of cleanliness and organize Cleaning Days in their neighborhoods*. “The awareness raised in small groups really makes people pay attention to the Puebla Limpia program. It reinforces what they see on TV or listen to on the radio and it motivates them to undertake a cleaning day, it also generates a greater commitment in adopting habits *…* becoming a large army of allied citizens who actively participate in Cleaning Marathons” (General Coordinator of Cleaning Marathons)*.

Regarding the level of information retention generated by the awareness meetings, in the short interviews, people were better able to define the proposed habits and recognized the importance of the two habits that were the hardest to change: fix and maintain the facade of the building and take out the trash in closed bags before the garbage truck passes. *“It’s something that the city government is organizing, to which we are invited to participate in, so that the city is clean, like taking out the garbage in tightly closed bags and giving a hand to paint” (Araceli, 52, housewife)*.

We must try to be clean, and we must clean the sidewalk, the yard, fix the façade, and take care of our dog when we take it out in the street, they are right (Amelia, age 30, elementary school education).

It was also interesting to note from the awareness meetings that there was a positive association with the work done by the City government that promoted this program, *“Until the government cares about this subject and us, we must be jointly responsible with them so that we better the city […] to keep it clean in front of the house, and take out the trash just before the garbage truck passes is not much” (Catherine, 30, elementary school education). “I congratulate and I’m glad that President Alcalá is doing this because before no politician was interested in the issue of cleanliness in the communities, and the City, and if they did, I did not hear about it (laughs)” (Consuelo, 53, housewife)*.

The target public had a favorable perception of the awareness meetings as a means of dissemination and persuasion for the Puebla Limpia program. They believed that in addition to entertaining they were educational, and the main advantages of the meetings were to expand and strengthen the information provided by traditional (mass) media and obtain new information about how to cooperate to keep the city clean.

*“It’s okay to come see us and motivate us to try to recover the values we have lost and it is proof that the government does not forget the outer communities” (Marisol, 44, middle school education). “It’s okay because many times you don’t remember what you see on TV or you do not pay attention, in contrast, with conversation, we had an hour to discuss our doubts […] I liked that he gave us tips on how to recycle trash” (Maribel, 20, high school education). “I like it because I talked with my neighbors, I learned things I did not know and the problems created by not keeping it clean […] also it is something creative, different from the usual, so one learns” (Henry, 56, technician)*. The meetings turned out to be very enriching and a highly motivating activity, not only educational, but bringing members of the community to work for a common goal.

We concluded that the target public knew the overall campaign by means of the mass media, that is to say the advertising used met its communication function. However, it failed to persuade the audience to take strong actions, such as modifying habits. Through alternative media, however, the target audience did remember the habits that the campaign proposed. They understood the importance of the subject and were motivated to take action in cleaning their environment.

In general, awareness meetings, as an alternative, worked for the attendees to remember cleaning habits. They gave importance to the issue, and there was a perceived benefit to enhance their environment from the information provided. A decision was taken to incorporate this new information into their daily lives; however, one could hardly talk of an immediate change in public habits.

### Partnership

In the program realized in 2008, alliances were made with mass media, businesses, schools, civic organizations, and religious organizations that expressed interest in the *Puebla Limpia* program to engage their cooperation in various cleanup activities. However, for the 2009 program, they tried to find allies in different district areas to help clean, such as locating community leaders who wanted to start a cleaning project with their neighbors (cohesion). From this situation, there were significant findings.

The positive leadership of the different districts generated cleaning movements in different zones of the city, which were successful in encouraging *Puebla Limpia* within their communities. This gave rise to social cohesion toward the program. *“In the districts where there is good leadership is where the cleaning project works best. It is there that neighbors motivate the others, and then they continue with their own initiative. Also among themselves they recognize the bad or the good actions of their neighbors. A small group of neighborhood leaders can move consciences, first in the spaces closest to theirs, and after some months they achieve to cover much larger areas *… *to extend the project to clean and maintain the cleanliness in a place where previously it has not functioned with negative leaders, who faced resistance” (General Coordinator of Cleaning Marathons). “The 2008 campaign had allied companies, but I think that with this 2009 campaign, its strength is that the allies are leading citizens of their communities. There is a vast army of allies and the citizens are precisely those who are actively involved in cleaning marathons. There’s a procedure to invite and engage them, and they are organized by themselves, the responsibilities are shared and they develop their projects together” (Marathon Coordinator for Lower Class Communities)*. Once again, we see that the most powerful force is that of the members of society.

### Policy

During the 2008 program, one of the most common feedback comments from people in focus groups was the proposal to implement fines and penalties for people who littered the city because they believed that if the authorities did not punish them, people cared little about the city being dirty. *“It is urgent to implement fines for littering; it is very sad as a little hand reaches out from the car window and throws garbage in the street and the traffic police do nothing”*. The threat of a fine, apparently as an incentive for people to avoid a negative or harmful behavior: *“We are a society that obeys the fines, for example, when they began to give out tickets for not wearing a seat belt, people began to use them” (Araceli, 52, housewife)*. City officials agreed with the policy approach:

Announcing the new policy was good, because it is reassuring to know that those who do not have good behavior at last are going to be fined or sanctioned. People actually disagree that we are dirty (General Coordinator for Awareness Meetings).

### Factors related to effectiveness

As demonstrated in the model, there are five factors that we found to be related to effectiveness: social, educational, economic, demographic, and cultural factors.

#### Social Cohesion

The most important process was initiating social cohesion. For example, districts that were divided and in conflict with neighbors that managed to overcome differences and unite for a cause.*“Puebla Limpia has allowed them to reunite, as it is a noble cause that pleases everyone and to which they have joined voluntarily. The Municipal Government gives materials: brooms, dustpans, trash bags, buckets, and paint, and the citizens give their labor” (General Coordinator for Puebla Limpia projects)*.

When neighbors define what their needs are and how to address them, when some motivate others, and when we develop networks for motivation and work, this is when there is a better result in the process of change, when we have finally achieved social cohesion (Mayor of Puebla).

Social cohesion and participation was an area that supported the motivation of the people and consequently the adoption of habits. This included the imitation of other people’s actions in some cases or repeating what others were doing. The joint participation of the people doing their best adoption of habits was a situation that could be seen in the marathons.

Some neighbors who were a little stubborn to cooperate and sometimes had to be pressured, were those who had their window boxes full of grass or trash, or lots of flying litter in front of their house … then we organized the neighbors and went door to door telling other neighbors that it was not worthwhile that some neighbors sweep in front of their houses or take out the garbage in cans if others did not, because the air, the garbage pickers and other people pass by and make a mess … then gradually they began to tell neighbors first to take out the trash on the new schedule because there were some neighbors who left their garbage cans outside all day and it looked bad … then they proposed several options; the first to pay someone to sweep the whole street and second that they rotate among themselves to sweep. In the end they decided to pay someone … but I think it was more because of social pressure. They knew that it was organized and they would feel out of place if they didn’t cooperate. (Mr. Flores, 50, leader of the community Prados Agua Azul).

#### Educational Factors

The poorest settlements in the city of Puebla were those whose inhabitants had the lowest level of schooling, and they were the most reluctant to accept the program. They had other priorities to attend to, the cleanliness of their streets was not a priority in their lifestyle, and they cared more that their basic needs were met first *“The level of education plays an important role in the adoption of habits, since normally when people have a higher educational preparation they more readily accept the importance of having a clean city. Nevertheless areas of low strata are found to claim that they pay taxes, and so it is the duty of the city municipal government to clean, in addition to first wanting the government to legitimize their land or pave the streets” (Coordinator for Awareness Meetings)*. It seemed the lower the educational level, the more they expected the government to do the job. People in this social class resisted most to change, even though it was for their own well-being.

#### Economic Factors

Those responsible for the cleanliness of the city recorded that the upper middle–class or upper-class neighborhoods did not need cleaning marathons, unlike housing complexes, government housing, or remote or lower class communities who had problems with trash in the streets, garbage in vacant lots or bus stops, graffiti, and dirty streets from food stalls among other things. *“The cleaning marathons were conducted in middle and low-class residential areas, and were not carried out in the communities of medium-high and high strata, like El Mirador, La Paz or any gated communities because there was really no problem with cleanliness, and priority was given to address the biggest problem areas presented. The greater economic and educational level reflected an increased awareness by throwing trash in proper receptacles” (General Coordinator of marathons)*.

#### Demographic Factors

Adults and especially elderly people proved extremely accepting of the Puebla Limpia marathons. *“Adults and elderly people were the most enthusiastic in organizing the cleaning marathons” (General Coordinator of Marathons)*. However, areas with higher population density had more problems with dirtiness *“The places where many people live, as housing complexes like the Margarita where 40 thousand inhabitants live, bus stops, where there is a lot of pedestrian traffic, or outside the markets, is where the most filth is generated” (Director of Operations, Collection Services)*.

#### Cultural Factors

The misinformation of the public caused false beliefs that inhibited the effectiveness of the program. For example, *that “the gullies are to throw the garbage in, or the false belief that I should throw my litter on the street, that’s why I pay my taxes, or the municipal government is going to take care of it” (Operational Director of Collection Services)*. Another factor was custom and the social dynamic. That was, *“people throw trash because it has always been done and no one says anything, take out the garbage however you want or when it is best for you because it convenient, or because no society can judge the act of the rest of society. The apathy towards social issues is common in Latin American countries *… *. “It will take more time to achieve change; it has been more than 30 years since people were asked to clean up” (Coordinator of Public Housing Marathons)*. We are used to delegating our civil responsibilities and expect the authorities to do the job for us, without cooperation. The truth is that a good citizen is always willing to work as a team member in order to improve his own life conditions.

### Effectiveness

The greatest indicator of effectiveness is the impact of *Puebla Limpia* on long-term cleanliness. We found that the program had a better effect in remote areas, where continuous and systematic work was carried out over almost 3 years into the program that the municipal government invested in cleaning, but also where residents organized to clean their spaces spontaneously, voluntarily, and continuously. “*There are remote areas that started by organizing a marathon and now every 15 days they organize to clean areas and have been doing so for months!*… *and of course they have already seen a significant change. There are other areas that have been cleaned by all the neighbors and then to keep them clean they imposed fees to pay a maintenance service for cleaning as in San Bartolo. San Bartolo is not the same now as it was two years ago” (Head of Public Housing Marathons)*. The improvement caught the eye, and showed a big difference when comparing the before and after images.

There were public housing complexes that achieved significant changes:

San Bartolo Housing Complex: this housing district had the characteristic of the organization of its inhabitants and leaders motivated to clean up the site, which resulted in cohesion on the concept: *“Their conscience awoke, it was dirty two years ago, and now we can see the change since you arrive to the place. The best part is that it stays clean. We organized a marathon in 2009, and they have done 24 marathons in a voluntary way. In addition a cleaning staff was hired that everyone contributes towards for maintenance of their area. To do this, each area has its own maintenance fees. In San Bartolo there are leaders who have led the change over the almost two years. They have also made their own rules of cleanliness of common areas” (Coordinator of Housing Complex Marathons)*. There was a difference between a dirty and a clean community, and members of the community were motivated to keep it this way.

Villa Frontera: this unit had problems with local organization, but on the part of Puebla Limpia, they managed to organize a group of residents and that led to the cleanliness of the place: *“This year has seen a perceptible change, substantive, although we cannot claim to be keeping it clean, it has been awarded the “Recognition of Merit” recognizing that much has been done even though it is not a totally clean place yet. It has an organized group of neighbors who have tried together to solve their problems of cleanliness and insecurity. The award has raised their self-esteem. They had spent two years without meeting as neighbors, but now they are united in an effort to improve their environment” (General Coordinator of Marathons)*.

La Margarita: this housing complex is a home for over 40,000 inhabitants, the largest in the city of Puebla, and had significant changes over this period of almost 3 years. In the first year, we could not observe changes due to its size; but in 2009, they took first place in the marathons thanks to good leadership, motivation, and organization of groups of people, who continued to organize cleaning marathons during the year: *“in 2008 the Margarita won the Merit Award, in 2009 they took first place. There is a constructive leadership on all matters pending, including cleaning, maintenance, and safety. Since Puebla Limpia started they have organized not only to clean up the site, but to improve the gardens, rebuild sidewalks, curbs, and remove graffiti. Cleaning marathons are on their own initiative, there is a constant process of rehabilitation of the complex, the people do their work, adapt the space, they each clean their areas and of course maintain them” (General Coordinator of Marathons)*.

Agua Santa: it was a remote complex with many cleaning problems; for this reason, the process of change was considered to be long term *“they began their process of change, however will take time till you can see something significant. The unit was completely deteriorated, plus there are no rules because nobody respects them. It is an area with problems of insecurity, however, there is good leadership, and the people are beginning to recognize this and are having ongoing cleanup work. If they continue, it will change like the Margarita, and will be clean, however difficult it may seem” (Coordinator of Housing Complex Marathons)*.

In general, all remote complexes that initiated cleaning marathons saw remarkable changes, as mentioned in the previous examples, which were those who continued the work of cleaning with their own initiative, by motivating people and from the role of different leaders. Even though the housing complexes that had cleaning marathons saw substantial changes, it was also clear that not all experienced the same change.

## Discussion

According to the premise that the effectiveness of the program depends on the involvement of people and the level of their participation and the change in behavior or habits in the population (14) with respect to the subject of cleaning the city, we conclude that in general, Puebla residents, the population program and program strategies increased the awareness of cleanliness of the City, which ultimately contributed leading to cleanliness becoming an important public issue. Campaign participants found that the proposed cleaning habits could contribute to improving their environment and they expressed an acceptance to carry them out.

The habit of “no littering”, or putting garbage in proper public receptacles was the most widely accepted because it required the least effort and had the maximum benefit. Residents felt that people threw garbage in the streets because of lack of education. While viewed as burdensome, the habit of “sweeping” was an action that grew in acceptance during the interventions according to the qualitative research. However, people had trouble accepting the habits of painting facades and taking out the trash just before the garbage truck passed due to effort, motivation, time, and money implied. Perhaps the most important indirect benefits brought by the Puebla Limpia program was to help establish a framework for citizen participation in city government, not only reinforcing citizens’ habits in cleaning but also construction of citizenship.

In this case study, we found that the effectiveness of the social marketing mix of a program thus depends on the complexity of what is required. People were not willing to adopt the habit of cleaning the facades of homes since it involved financial expense or taking out the trash just before the truck passes because it did not coincide with being home. In other words, if the habit implies a high social cost, it is more difficult for people to adopt it.

In addition to complexity, another factor that influences the effectiveness of the social marketing mix is the organization of the neighbors as a key factor for change. In *Puebla Limpia*, the Cleaning Marathons were motivated by monetary incentives, which then provoked cohesion with neighbors toward the common goal of winning one of the categories. The goal united neighbors to clean voluntarily and continuously and to pressure those who did not do their part or made things dirty.

With respect to media, Freimuth (15) mentions that the effectiveness of the campaign depends on the characteristics of the audience, complexity of the issue, and exposure to the message through multiple channels. The *Puebla Limpia* campaign demonstrated that the effectiveness of mass media depended on the content transmitted, its relevance to the lives of the people, frequency and exposure to the message through multiple channels, the context in which the message was received, the mood the people were in, the social influences, and the amount of time necessary to keep the topic in their memory.

Action Research as a methodology played an important role in this study. The research investigator had dual roles, both to take action and to create knowledge or theory about that action that might be applied in other cases. Distinct from traditional research approaches that aim at only creating knowledge, the action researcher was involved with two roles: as a consultant and a researcher.

Finally, we conclude that the process of change starts with a lack of awareness and then the decision to change begins. The results of this program affirm that in order for change to take place, people need to know what to do and how to do it. This is followed by achieving a change of habit by repetition, and then finally the maintenance phase of the new habit. So, too, there is a process of change on different levels according to different circumstances and due to different long-term strategies.

## Conflict of Interest Statement

The authors declare that the research was conducted in the absence of any commercial or financial relationships that could be construed as a potential conflict of interest.
